# Dataset of food delivery app users at a public university: Insights into digital transformation

**DOI:** 10.1016/j.dib.2023.109161

**Published:** 2023-04-20

**Authors:** Ha Hoang, Ngoc Tuan Chau, Trinh Le Tan

**Affiliations:** a Faculty of Business Administration, University of Economics, The University of Danang, Danang City, Vietnam; b School of Business IT & Logistics, RMIT University, Melbourne, Australia; c Faculty of Statistics and Informatics, University of Economics, The University of Danang, Danang City, Vietnam; d Business Department, FPT University, Da Nang 550000, Vietnam

**Keywords:** Food delivery apps, Digital transformation, Continued intention, Electronic word-of-mouth, Vietnam

## Abstract

The food delivery apps (FDAs) have facilitated the connection between food service providers and consumers, enabling online ordering through smartphones and offline delivery in Vietnam. The Covid-19 pandemic has significantly impacted the food and beverage industry, accelerating the process of digital transformation and promoting sustainability through online-to-offline service. The usage of FDAs amongst consumers has exhibited a discernible escalation, primarily attributable to its ability to expedite the delivery of food in a hassle-free and convenient manner. Given the ongoing pandemic and the swift increase in demand for online food ordering services, particularly among the younger demographic, it has become imperative to comprehend the drivers that impel consumers to adopt these applications.

This article aims to present a dataset pertaining to the decision-making factors that university students in Danang, Vietnam, take into account when they use FDAs and express positive feedback about them on the internet. The survey was conducted between September 2022 and January 2023 and gathered 346 usable responses. The results provide novel perspectives on the adoption of FDAs by university students, which is an emerging technology in the food and beverage sector. This dataset could be useful to various stakeholders, such as service providers, small and medium enterprises (SMEs), and vendors operating on these platforms, as it can help them acquire valuable insights into their customers' preferences and behavior. In addition, the dataset can serve as a basis for conducting comparative research in different universities or countries.

**Specifications Table**

**Table:** 

Subject	Management of Technology and Innovation
Specific subject area	Food Delivery App Usage Behavior
Type of data	Table, Figure, MS Excel file
How data were acquired	The data was collected through two methods: online using Google Forms and in-person using paper surveys. The data analysis was conducted using various instruments, including Excel, SPSS, and SMARTPLS.
Data format	Raw, Analyzed
Description of data collection	Between September 2022 and January 2023, data was collected using a non-probability sampling method by distributing questionnaires to students via Google Forms link or paper survey. Missing values were identified and addressed accordingly. Out of the 438 responses collected, 346 were considered valid after removing answer sheets with missing or identical responses, resulting in a response rate of 78.99%.
Data source location	City/Town/Region: Da Nang Country: Vietnam Coordinates: 16.0544° N, 108.2022° E
Data accessibility	Repository name: Mendeley Data Data identification number: 10.17632/z8vrr5hk33.2 Direct URL to data: http://dx.doi.org/10.17632/z8vrr5hk33.2

## Value of the Data

• Researchers can easily have access to the raw data, and it is already prepared for their use.
• The information provided by the data exposes the connections among various factors such as task-technology fit (TTF), performance expectations, ease of use, social influences, perceived cost, confirmation, quality of information, habit, pandemic situation promotion, satisfaction, continuance of use, and electronic word-of-mouth (e-WOM) of students in their usage of food delivery apps (FDAs).
• The data has the potential to offer valuable insights into the future trends and advancements in society, as students are often considered to be the future workforce and potential leaders.
• The dataset offers an opportunity for conducting comparative studies at different universities or countries. This valuable resource can serve as a starting point for exploring similarities or differences in customer behavior in the context of FDAs across various cultural and socio-economic settings.
• The dataset is therefore an important resource for researchers, food industry professionals, and policymakers who are interested in the intersection of technology, food, and consumer behavior.

## 1 Objectives

FDAs are acquiring popularity worldwide, particularly in metropolitan locations where customers need quick, convenient, and diverse food ordering alternatives. During the Covid-19 outbreak, when contactless food delivery becomes critical, these apps provide customers and food suppliers a safer alternative. The dataset procured from a public university situated in Danang, Vietnam, bears substantial significance in scrutinizing the intricate decision-making procedures that influence the utilization of FDAs among university students, who constitute an increasingly expanding consumer segment. The publication of this dataset article is driven by the recognition that data sharing is an essential element of contemporary scientific research. Sharing data promotes transparency, replicability, collaboration, and efficiency, and makes scientific knowledge more accessible, inclusive, and democratic [1]. The dataset on FDA usage among university students offers valuable insights for service providers, SMEs, and platform sellers to understand consumer behavior. This can aid in informed business decisions, improved marketing, and better consumer experiences in the food and beverage industry's digital transformation. The dataset also provides a basis for comparative research, expanding knowledge of FDAs usage.

## 2 Data Description

The focus of this article is to provide a descriptive analysis of quantitative data related to the factors that influence the sustained usage and positive electronic word-of-mouth (eWOM) regarding Food Delivery Apps (FDAs) among Vietnamese university students. The data collection method involved the utilization of a survey approach, which included a questionnaire comprising 56 items that employed a five-point Likert scale to assess the participants' responses. The survey utilized in this study comprises 13 research variables and 56 measurement items. These items were adapted from previous research and are listed in Table 1, along with their corresponding constructs and measurement items' sources. The responses were assigned a numerical value of 5 for “strongly agree”, 4 - “agree”, 3 - “neutral”, 2 - “disagree” and 1 - “strongly disagree”.

**Table 1:** Constructs and measurement items.

Constructs		Measurement items	Sources
Task-Technology Fit (TTF)	TTF1	The functions of FDAs support the management of ordering and receiving foods.	[2,3]
	TTF2	The functions of FDAs fully meet my requirements for ordering and receiving foods.	
	TTF3	The functions of FDAs help me to keep my distance from others to protect myself.	
	TTF4	Overall, the FDA functions completely met my need to order food online.	

Performance expectancy (PE)	PE1	I find the FDAs very useful for my daily life.	[4]
	PE2	Using the FDAs gives me many opportunities to buy a variety of food items that are convenient for me.	
	PE3	Using the FDAs helps me shorten the purchasing process.	
	PE4	I can save time when using the FDAs to buy food.	

Ease-of-use (EU)	EU1	It's easy to use FDAs	[5]
	EU2	The ordering process on FDAs was very easy for me.	
	EU3	FDAs are very clear and easy to understand	
	EU4	Learning how to use FDAs is easy.	

Perceived cost (PC)	PC1	Food on FDAs is reasonably priced.	[4,5]
	PC2	The quality of food on FDAs corresponds to the money spent	
	PC3	Currently, FDAs offer good prices.	
	PC4	I believe I can save money using FDAs	
	PC5	FDAs save me money by comparing prices between different stores.	

Continued Intention to use (CI)	CI1	I look forward to using FDAs more in the future.	[6,7]
	CI2	I plan to use FDAs in the future.	
	CI3	If there is a chance, I will use FDAs to buy foods.	
	CI4	I will try to use FDAs.	
	CI5	I will frequently use FDAs.	

Confirmation (CO)	CO1	My experience using FDAs is great as expected.	[8,9]
	CO2	Overall most of my expectations when using the FDAs were met.	
	CO3	The services provided by FDAs are better than my expectations.	
	CO4	My expectations for FDAs were correct.	

Information quality (IQ)	IQ1	FDAs allows me to search and get the information I need quickly and efficiently.	[10]
	IQ2	The information provided by FDAs is accurate and reliable.	
	IQ3	The information provided by FDAs is easy to understand and clear.	
	IQ4	FDAs helps me get real-time updates.	

Satisfaction (SA)	SA1	I am completely satisfied when I buy food on FDAs.	[11]
	SA2	I think buying food on FDAs is the right decision.	
	SA3	I am very satisfied with the service provided by FDAS.	
	SA4	I am very satisfied with the food purchased on FDAs.	

Ewom (EW)	EW1	I am willing to provide positive information about FDAs to internet users.	[12,13]
	EW2	I am ready to actively recommend FDAs to relatives and friends on social networks.	
	EW3	I am willing to share positive information about FDAs on the internet.	
	EW4	I'm open to positive reviews and discussions about FDAs.	
Habit (HT)	HT1	Buying food through FDAs seems to have become a habit for me.	[11]
	HT2	I can't stop using the FDAs.	
	HT3	I need to use FDAs to buy food.	
	HT4	Using FDAs gradually became more natural to me.	

Pandemic Situation (SIT)	SIT1	During the COVID-19 pandemic, many grocery stores are closed.	[14,15]
	SIT2	During the COVID-19 pandemic, there are significant health risks associated with going to grocery stores.	
	SIT3	During the COVID-19 pandemic, shopping for food via FDAs is a trend.	
	SIT4	During the COVID-19 pandemic, the epidemic prevention regulations made it very difficult for me to buy food in the traditional way.	
	SIT5	During the COVID-19 pandemic, buying food through FDAs helps reduce contact and enforce distancing regulations.	

Promotion (PM)	PM1	There are many promotions when using FDAs.	[16]
	PM2	I am very interested in promotions and offers when using FDAs.	
	PM3	Using FDAs brings me many benefits thanks to the accompanying incentives.	
	PM4	I like to search for promotions at various online stores when buying food on FDAs.	

Social influence (SI)	SI1	People who are important to me think that I should use FDAs for buying food.	[5]
	SI2	People who influence my behavior think I should use FDAs for buying food.	
	SI3	People I respect think that I should use FDAs for buying food.	
	SI4	I use FDAs because my friends use it a lot.	
	SI5	I use FDAs because my relatives use it a lot.	

The collected raw data is saved in an Excel file that contains 347 rows and 61 columns. The first row indicates the measurement items and surveyor characteristics, while rows 2 to 347 represent the data of each of the 346 participants in the survey. Columns 1 to 56 contain the respondents' information related to the research variables, while columns 57 to 61 contain data related to the demographic characteristics of the survey respondents.

In the process of data analysis, five tables and one figure were compiled to describe the characteristics of the respondents, provide descriptive statistics, and evaluate the reliability and validity of the instrument. The dataset used for statistical analysis comprised of 346 participants, and the results have been presented in Table 2, Table 3, Table 4, Table 5 as follows.

**Table 2:** Demographic information.

Variable	Characteristic	N	Percentage
Gender	Male	70	20.2
	Female	276	79.8

Using time	Less than 3 months	91	22.8
	From 3 months to less than 1 year	83	23.7
	From 1 year to less than 2 years	92	27.2
	From 2 years	80	26.3

FDAs usage frequency	Rarely	58	16.8
	Occasionally	144	41.6
	Frequently	101	29.2
	Always	43	12.4

Income	Less than 5 million VND (approximate 212 USD)	235	67.9
	From 5 million VND to less than 10 million VND	64	18.5
	Over 10 million VND	47	13.6

Number of internet-connected devices	1	231	66.8
	2	93	26.9
	≥ 3	22	6.4

**Table 3:** Mean, standard deviation, Skewness, and Kurtosis.

Construct	Item	N	Mean	Std. Deviation	Skewness	Kurtosis
TTF - Task Technology fit	TTF1	346	3.99	.978	-1.211	1.462
	TTF2	346	4.02	.928	-1.026	1.065
	TTF3	346	3.84	.969	-0.647	.163
	TTF4	346	3.80	.967	-0.700	.151

PE - Performance expectancy	PE1	346	3.94	.905	-0.865	.794
	PE2	346	3.93	.944	-0.855	.590
	PE3	346	3.92	.964	-0.730	.152
	PE4	346	3.98	.994	-0.850	.180

HT - Habit	HT1	346	3.27	1.124	-0.101	-0.701
	HT2	346	3.25	1.170	-0.084	-0.843
	HT3	346	3.09	1.204	-0.073	-0.918
	HT4	346	3.12	1.226	-0.091	-0.943

SI - Social influence	SI1	346	3.22	1.058	-0.085	-0.607
	SI2	346	3.27	1.033	.007	-0.580
	SI3	346	3.24	1.061	-0.017	-0.584
	SI4	346	3.27	1.155	-0.232	-0.764
	SI5	346	3.16	1.147	-0.001	-0.847

EU - Ease of use	EU1	346	3.90	.945	-0.929	.795
	EU2	346	3.96	.884	-0.859	.951
	EU3	346	3.98	.886	-0.765	.608
	EU4	346	3.89	.904	-0.729	.335

PC - Perceived cost	PC1	346	3.53	.920	-0.299	-0.196
	PC2	346	3.53	.917	.073	-0.326
	PC3	346	3.68	.893	-0.264	-0.309
	PC4	346	3.41	1.071	-0.228	-0.593
	PC5	346	3.75	.986	-0.611	-0.092

PM - Promotion	PM1	346	3.96	.835	-0.590	.210
	PM2	346	4.13	.927	-1.112	1.128
	PM3	346	3.96	.879	-0.648	.187
	PM4	346	3.96	.932	-0.828	.622

SIT - Pandemic situation	SIT1	346	3.91	.908	-0.781	.509
	SIT2	346	4.02	.894	-0.792	.465
	SIT3	346	4.08	.847	-0.787	.437
	SIT4	346	3.99	.891	-0.780	.479
	SIT5	346	4.11	.882	-1.113	1.522

CI - Continued intention to use	CI1	346	3.79	.862	-0.574	.594
	CI2	346	3.87	.869	-0.738	.702
	CI3	346	4.01	.851	-0.635	.283
	CI4	346	3.62	.915	-0.326	-0.082
	CI5	346	3.62	.934	-0.365	-0.075

IQ - Information quality	IQ1	346	3.89	.845	-0.768	.948
	IQ2	346	3.74	.886	-0.247	-0.315
	IQ3	346	3.89	.796	-0.489	.501
	IQ4	346	3.93	.847	-0.564	-0.027

CO - Confirmation	CO1	346	3.65	.818	-0.226	.056
	CO2	346	3.76	.867	-0.393	-0.060
	CO3	346	3.60	.839	-0.095	-0.132
	CO4	346	3.66	.857	-0.187	-0.315

SA - Satisfaction	SA1	346	3.67	.846	-0.290	-0.045
	SA2	346	3.64	.907	-0.239	-0.075
	SA3	346	3.76	.837	-0.354	.067
	SA4	346	3.67	.889	-0.176	-0.356

EW - Electronic word of mouth	EW1	346	3.82	.797	-0.429	.243
	EW2	346	3.88	.825	-0.456	.082
	EW3	346	3.77	.885	-0.610	.572
	EW4	346	3.81	.830	-0.428	.049

**Table 4:** Assessment of measurement items.

Constructs/items	Outer loadings	Cronbach's Alpha	Composite Reliability	AVE	VIF
CI		0.865	0.902	0.649	
CI1	0.821				2.142
CI2	0.815				2.221
CI3	0.787				2.026
CI4	0.814				2.132
CI5	0.791				1.958
CO		0.871	0.912	0.721	
CO1	0.89				2.742
CO2	0.835				2.216
CO3	0.847				2.138
CO4	0.824				1.913
EU		0.873	0.913	0.726	
EU1	0.851				2.191
EU2	0.9				2.837
EU3	0.863				2.283
EU4	0.789				1.785
EW		0.853	0.901	0.694	
EW1	0.841				2.024
EW2	0.845				2.061
EW3	0.845				2.053
EW4	0.801				1.769
HT		0.907	0.935	0.782	
HT1	0.882				2.821
HT2	0.898				3.100
HT3	0.876				2.765
HT4	0.882				2.838
IQ		0.835	0.889	0.668	
IQ1	0.794				1.730
IQ2	0.842				1.851
IQ3	0.819				1.847
IQ4	0.813				1.754
PC		0.875	0.909	0.668	
PC1	0.864				2.564
PC2	0.814				1.994
PC3	0.84				2.284
PC4	0.801				1.946
PC5	0.765				1.781
PE		0.806	0.873	0.633	
PE1	0.819				1.800
PE2	0.769				1.675
PE3	0.831				1.882
PE4	0.761				1.603
PM		0.854	0.901	0.695	
PM1	0.836				1.994
PM2	0.849				2.143
PM3	0.842				2.004
PM4	0.807				1.749
SA		0.88	0.918	0.736	
SA1	0.889				2.732
SA2	0.853				2.293
SA3	0.841				2.069
SA4	0.847				2.171
SI		0.907	0.93	0.728	
SI1	0.868				2.897
SI2	0.882				3.127
SI3	0.864				2.762
SI4	0.815				2.413
SI5	0.835				2.578
SIT		0.876	0.909	0.667	
SIT1	0.844				2.070
SIT2	0.86				2.305
SIT3	0.769				1.871
SIT4	0.777				1.827
SIT5	0.829				2.338
TTF		0.827	0.885	0.659	
TTF1	0.861				3.085
TTF2	0.834				2.740
TTF3	0.714				1.333
TTF4	0.831				1.703

**Table 5:** Heterotrait-Monotrait Ratio (HTMT).

	CI	CO	EU	EW	HT	IQ	PC	PE	PM	SA	SI	SIT	TTF
CI													
CO	0.818												
EU	0.755	0.696											
EW	0.764	0.792	0.745										
HT	0.701	0.776	0.493	0.583									
IQ	0.862	0.822	0.767	0.776	0.569								
PC	0.778	0.779	0.641	0.686	0.788	0.734							
PE	0.795	0.693	0.786	0.718	0.527	0.813	0.676						
PM	0.746	0.661	0.862	0.738	0.355	0.823	0.645	0.767					
SA	0.877	0.934	0.682	0.791	0.774	0.807	0.784	0.691	0.664				
SI	0.686	0.742	0.447	0.56	0.903	0.593	0.785	0.51	0.41	0.771			
SIT	0.708	0.611	0.765	0.768	0.353	0.787	0.59	0.803	0.856	0.605	0.375		
TTF	0.76	0.668	0.781	0.74	0.433	0.811	0.592	0.888	0.785	0.689	0.43	0.826	

Information on the characteristics of survey respondents who have used FDAs is presented in Table 2, covering gender, duration of use, usage frequency, income level, and the number of internet-connected devices. The data shows that females constitute the majority of respondents. A majority of the respondents have been using FDAs for over a year, and occasional usage is the most common frequency reported. The majority of respondents earn less than 5 million VND and use only one internet-connected device.

According to the data presented in Table 3, which contains the Mean and Standard Deviation values of measurement items after undergoing processing with SPSS software, we conducted an assessment of the data's conformity by analyzing its Skewness and Kurtosis. Our evaluation determined that all the data points remained within the acceptable range for both measures, as specified in Table 3. Normality is evaluated through two primary components, namely skewness and kurtosis. Hair Jr, Hult, Ringle, Sarstedt, Danks and Ray [17] put forth critical values of ±2.58 for skewness and kurtosis at a significance level of 1%. The mean, standard deviation, skewness, and kurtosis values of the measurement items are presented in Table 3. Notably, all measurement items exhibit skewness values ranging from -1.211 to 0.73 and kurtosis values ranging from -0.943 to 1.462, which fall within the acceptable range for normality. Therefore, the data distribution can be deemed normal.

**Fig. 1:**
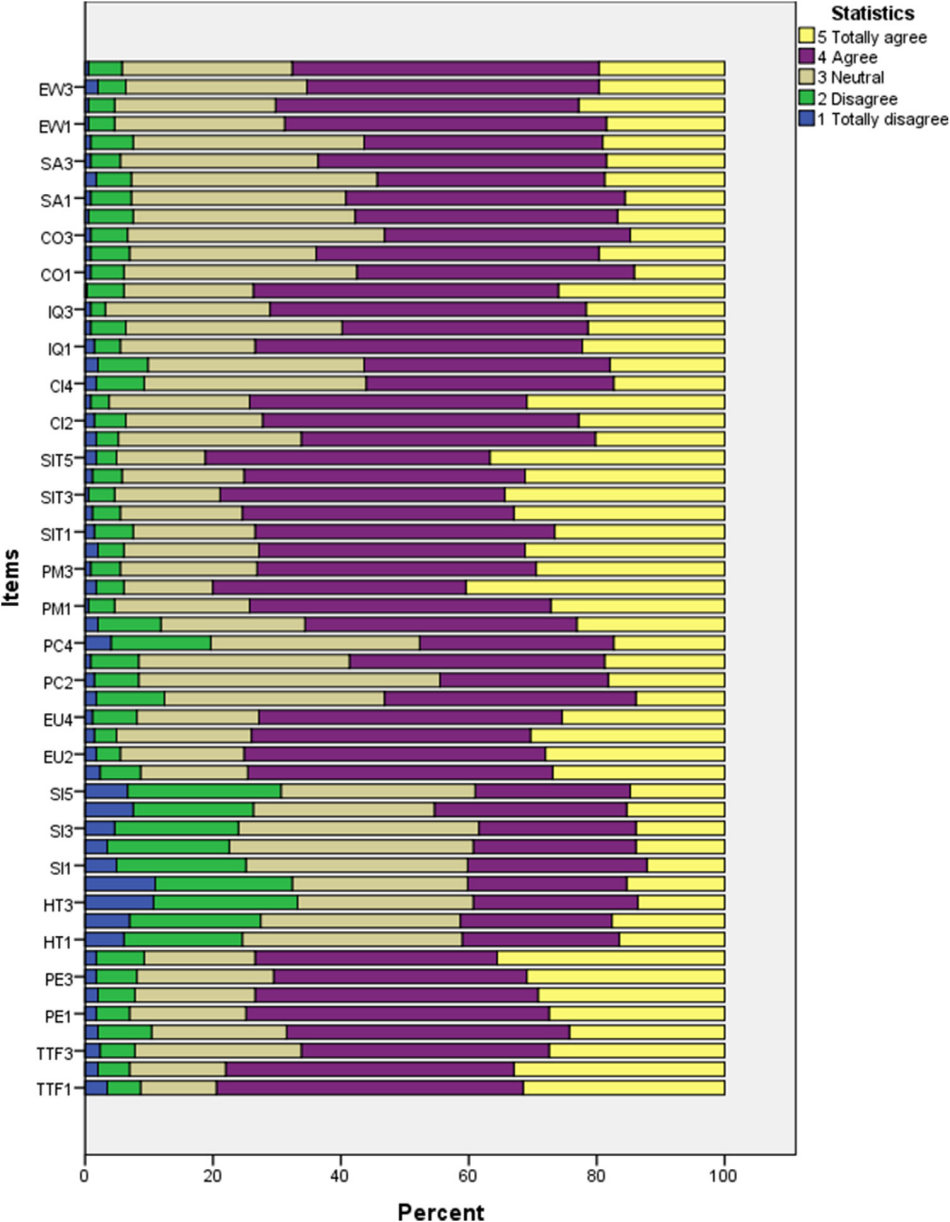
Relative distribution of responses to the survey scales.

The distribution of responses to the survey scales is depicted in Fig. 1 above. The visualization indicates that the habit (HT) and social influence (SI) scales received the highest proportion of disagree responses.

Reflective measurement models require the assessment of three different criteria, which are internal consistency reliability, convergent validity, and discriminant validity. Table 4 presents the results for Composite Reliability (CR) and Cronbach's alpha (CA), as well as the Average Variance Extracted (AVE) of all sub-constructs. The data collected meets the requirements for internal consistency reliability as all constructs have CR values above 0.7 (ranging from 0.873 to 0.935) and Cronbach's Alpha values also exceed 0.7.

The convergent validity has been evaluated by looking at two criteria: outer loadings and the average variance extracted (AVE). Table 5 below shows that all measurement items have outer loadings greater than the threshold of 0.6 recommended by Hair Jr, Hult, Ringle, Sarstedt, Danks and Ray [17]. The AVE, which measures the common variance in a construct, was found to be at least 0.5 for all thirteen constructs, with AVE values ranging from 0.633 to 0.782. These results show that there is an adequate convergent validity for all the constructs.

The assessment of discriminant validity using the HTMT criterion, as described by Hair Jr, Hult, Ringle, Sarstedt, Danks and Ray [17], is presented in Table 5. This criterion is used to determine how much a particular construct differs from other constructs by empirical standards. Our study utilized the threshold value of 0.9, as defined by Henseler, Ringle and Sarstedt [18], to evaluate the potential issue of discriminant validity in cases where the HTMT values exceed this limit. Our findings indicate that while the majority of the constructs we examined demonstrated satisfactory discriminant validity as per this criterion, the constructs of pandemic situation and habit, as well as satisfaction and confirmation, exhibited slightly higher HTMT values of 0.903 and 0.934, respectively. To further evaluate the discriminant validity of our constructs, we conducted a bootstrapping procedure with 5000 resamples and constructed a confidence interval. Our analysis revealed that the empirical 95% confidence interval did not include the value of 1, indicating sufficient discriminant validity as per Henseler, Ringle and Sarstedt [18]. Therefore, despite the higher HTMT values observed for some of the constructs, our findings suggest that there are no significant issues with discriminant validity in our study.

### 2.1 Limitations of the Dataset

Although the sample of 346 university students in a public university may provide valuable insights into the consumption patterns of a particular group, it is crucial to acknowledge that its generalizability to the broader population of Da Nang, Vietnam, may be limited. This is particularly pertinent to FDAs, given the significance of this group as a key customer demographic because of their proclivity for technology and their connectedness. The sample's representativeness is circumscribed by its focus on university students, whose consumption patterns may not align with those of other age groups or socioeconomic classes in the city. Moreover, the sample may lack diversity, given its exclusive inclusion of individuals attending a public university.

To mitigate these limitations, it is recommended that researchers interested in using this dataset for further investigations expand the sample size to include a more diverse range of participants. Specifically, incorporating individuals from various age groups, socioeconomic classes, and universities within Vietnam, or even from other countries, may lead to a more comprehensive understanding of consumption patterns in using FDAs. Furthermore, leveraging this dataset in comparative analyzes with other datasets from diverse contexts could help to provide additional insights into the subject matter at hand. Such measures would likely contribute to a more nuanced and robust interpretation of the findings, which could advance the literature in this domain.

## 3 Experimental Design, Materials and Methods

The goal of gathering this data set was to provide a foundation for any investigations looking into Vietnam's digital transformation process. For SMEs to have a more effective digital transformation strategy, they must understand their customers. The food and beverage industry is witnessing heightened competition, driving service companies to prioritize digital transformation in the wake of the Covid-19 pandemic. This research presents a dataset shedding light on the determinants of food delivery app usage among students in a Vietnamese public school. Two key reasons why data is collected on students are: firstly, students tend to be early adopters of new technologies and readily embrace new products and services. Therefore, analyzing their behavior and preferences can furnish companies and organizations with valuable insights for devising effective strategies and maintaining competitiveness. Secondly, students are the forthcoming leaders and decision-makers of society, hence it is essential to comprehend their values, beliefs, and attitudes toward diverse issues and phenomena to shape policies and initiatives that cater to their needs and aspirations.

In this study, the TAM, TTF, and ECM models are integrated with recently emerged factors in technology acceptance research, such as promotion, electric word-of-mouth, and pandemic situation. It is widely recognized in the academic community that relying on a single model alone is inadequate to explain human behavior, and therefore it is imperative to extend existing models and integrate new factors in order to enhance the predictive capability of such models [19]. The survey questions presented in Table 1 were developed based on previous relevant research [2], [3], [4], [5], [6], [7], [8], [9], [10], [11], [12], [13], [14], [15], [16]. We employed a mixed-mode approach to data collection, including both online and face-to-face methods involving the distribution of questionnaires. Prior to participating in the survey, all participants were informed about the research's objective and provided with informed consent. We implemented several measures for quality check, including a data screening process after data collection. This involved examining unengaged responses, identifying outliers, and assessing the normality assumption of data distribution using two components - skewness and kurtosis. Furthermore, the authors have implemented various measures to ensure that the respondents answer the online survey accurately. Clear instructions were provided, including guidance on how to answer questions, to minimize confusion and improve response accuracy. A pre-test was conducted to identify and resolve any issues with the survey, such as unclear or confusing questions, before administering it to the full sample. Additionally, the study's objectives were clearly communicated to participants, and confidentiality and anonymity were guaranteed, reducing the impact of social desirability bias. After removing answer sheets with missing or identical responses, 346 responses were considered valid out of the 438 responses collected, resulting in a response rate of 78.99%.

We utilized the SPSS software to conduct descriptive analysis on the collected data, which is presented in Table 2 showcasing demographic information. We assessed the data's conformity by inspecting Skewness and Kurtosis, and determined that all the data points remained within the acceptable limits for Skewness, Kurtosis, as specified in Table 3.

Fig. 1 was drawn using the SPSS software. Subsequently, we utilized SMARTPLS software to assess the collected data. The computed Composite Reliability (CR) and Cronbach's alpha (CA) values, along with the Average Variance Extracted (AVE) scores for each sub-construct, are presented in Table 4. All the outer loadings in Table 4 meet the criteria of being greater than 0.7 [20]. Furthermore, Table 5 elaborates on the discriminant validity of HTMT.

In general, this section provides a comprehensive overview of the research design and the procedures used to conduct the study, which is essential for other researchers to replicate the study and build upon its findings.

## Ethics Statements

We requested all survey participants for their agreement, which we received through a statement outlining the nature and aim of the survey. Furthermore, we ensured the respondents' anonymity, so that no personal information could be traced back to them.

The study was conducted in accordance with the Declaration of Helsinki, and approved by the Ethics Committee of Science and Technology Development Department—The University of Danang, Vietnam (Approval code 14/HD-KHCN-2021, Approval date: December 1st, 2021).

## CRediT Author Statement

**Ha Hoang:** Conceptualization, Investigation, Data curation, Software, Original draft preparation; **Chau Ngoc Tuan:** Methodology, Validation, Original draft preparation; **Trinh Le Tan:** Writing – review & editing.

## Declaration of Competing Interest

The authors declare that they have no known competing financial interests or personal relationships that could have appeared to influence the work reported in this paper.
